# Nanocomposites of Silicon Oxides and Carbon: Its Study as Luminescent Nanomaterials

**DOI:** 10.3390/nano13071271

**Published:** 2023-04-04

**Authors:** Gabriel Omar Mendoza Conde, José Alberto Luna López, Zaira Jocelyn Hernández Simón, José Álvaro David Hernández de la Luz, Karim Monfil Leyva, Jesús Carrillo López, Haydee Patricia Martínez Hernández, Erick Gastellóu Hernández, Dainet Berman Mendoza, Javier Flores Méndez

**Affiliations:** 1 Centro de Investigaciones en Dispositivos Semiconductores (CIDS-ICUAP), Benemérita Universidad Autónoma de Puebla (BUAP), Col. San Manuel, Cd. Universitaria, Av. San Claudio y 14 Sur, Puebla 72570, Mexico; 2 Departamento de Ingeniería Eléctrica y Electrónica, Instituto Tecnológico de Apizaco (ITA), Fco I Madero s/n, Barrio de San José, Apizaco 90300, Mexico; 3 División de Sistemas Automotrices, Universidad Tecnológica de Puebla (UTP), Puebla 72300, Mexico; 4 Departamento de Investigación en Física, Universidad de Sonora (UNISON), Hermosillo 83000, Mexico; 5 Facultad de Ciencias de la Electrónica (FCE), Benemérita Universidad Autónoma de Puebla (BUAP), Col. San Manuel. Cd. Universitaria, Av. San Claudio y 18 Sur, Puebla 72570, Mexico

**Keywords:** luminescence, nanocomposites, carbon nanotubes, graphene oxide, silicon rich oxide, porous silicon

## Abstract

In this work, hybrid structures formed by nanostructured layers, which contain materials, such as porous silicon (PSi), carbon nanotubes (CNTs), graphene oxide (GO), and silicon-rich oxide (SRO), were studied. The PSi layers were obtained by electrochemical etching over which CNTs and GO were deposited by spin coating. In addition, SRO layers, in which silicon nanocrystals are embedded, were obtained by hot filament chemical vapor deposition (HFCVD) technique. Photoluminescence (PL) spectra were obtained from the hybrid structures with which a comparative analysis was completed among different PL ones. The SRO layers were used to confine the CNTs and GO. The main purpose of making these hybrid structures is to modulate their PL response and obtain different emission energy regions in the PL response. It was found that the PL spectra of the CNTs/SRO and GO/SRO structures exhibit a shift towards high energies compared to those obtained from the PSi layers; likewise, the PSi/CNTs/SRO and PSi/GO/SRO structures show a similar behavior. To identify the different emission mechanisms originated by PSi, GO, CNTs, and SRO, the PL spectra were deconvolved. It was found that the Psi/CNTs/SRO and Psi/GO/SRO structures exhibit a PL shift in respect to the PSi layers, for this reason, the modulation of the PL emission of the structures makes these hybrid structures promising candidates to be applied in the field of photonic and electroluminescent devices.

## 1. Introduction

The discovery of photoluminescence (PL) in porous silicon (PSi) [1] attracted great interest in the potential implementation of silicon in photonic applications with the purpose of developing integrated optoelectronic devices made entirely of silicon. The intense PL emission in PSi is explained by the electronic quantum confinement in the nanostructures produced in the material, therefore, obtaining a nanostructured material is essential to improve the radiative emission phenomena.

In addition to the PL emission, the PSi has been extensively studied due to its unique characteristics, such as its wide specific area in a small volume, pore size control, biocompatibility, and its compatibility with silicon manufacturing technologies [2,3,4,5,6]. There are numerous techniques for processing crystalline silicon and to obtain the Psi. One of technique is the electro-anodization technique, which consists of immersing a crystalline silicon wafer in an electrolytic solution and passing a current through it for a specific time [1]. The physical effect that occurs in the silicon wafer is the formation of pores with different sizes ranging from micrometer to nanometer scales.

As previously mentioned, the use of silicon-based nanostructured materials is indispensable to acquire substantial improvements in their photonic application. A material of great technological interest in silicon optoelectronic research is silicon-rich oxide (SRO), which has been reported on multiple occasions as a material with nanostructures intrinsically formed within it. An outstanding characteristic of the SRO is that it facilitates the formation of silicon nanocrystals (Si-ncs) embedded in a SiO_2_ matrix [7], where electron quantum confinement is generated due to low-dimensional effects, leading to an efficient light emission [8]. Apart from the effects related to quantum confinement in nanostructures, it has been reported the presence of intrinsic defects in the SRO films that favor the radiative emission [9,10].

Although the two previously mentioned materials (PSi, SRO) are among the most promising alternatives in the field of silicon-based light emission, there are still several drawbacks to overcome to be fully applied to electroluminescent devices. In this context, in the present research work, as a previous step to manufacture silicon-based electroluminescent devices, the incorporation of carbon-based nanostructured materials (carbon nanotubes and graphene oxide) is proposed due to the multiple optical and electrical properties that they offer as it has been reported in this type of nanostructures [11,12,13], with the main purpose that these ones will improve the radiative emission effects in the aforementioned silicon based materials. These radiative emissions can be suitably studied by means of the PL and its modulation. 

As a justification of why low-dimensional carbon-based nanomaterials, such as zero-dimensional fullerenes, one-dimensional carbon nanotubes (CNTs), nanodiamond films, and two-dimensional (2D) graphene oxide (GO), are used, it is because they are promising materials for manufacturing optoelectronic devices, such as sensors, field emitters, and light-emitting applications, according to what has been reported in numerous studies [11]. Consequently, luminescent carbon nanostructures are expected to achieve high-efficiency PL in light-emitting devices [12]. 

The CNTs can be divided into three main categories according to their structural, mechanical, and electronic properties. The electronic properties of CNTs are strongly influenced by small structural variations, in particular, their metallic and semiconducting nature is determined by the diameter and helicity (chirality) of the carbon atoms of the CNTs. All this makes them suitable for improving the functionalization of existing materials, which is possible to create novel systems, such as semiconductor heterostructures. 

On the other hand, the 2D-GO is a carbon-based material that can be used in graphene-based optoelectronic devices, especially for biomedical applications, due to its unique electronic properties and large specific surface area [14,15]. Since graphene has no band gap as an intrinsic material, it is not expected to exhibit PL due to the relaxation of excited carriers; however, when it is functionalized, such as graphene oxide, it has a band gap and exhibits PL [16]. Therefore, GO can be used to form new optoelectronic devices where the presence of this material may improve the optical properties. In this work, we proposed two novel structures conformed by the Si-n/PSi/CNTs/SRO and Si-n/PSi/GO/SRO configurations. These nanostructured systems showed interesting photoluminescent properties based on the quantum effects due to their reduced dimensions. Therefore, such effects are expected to cause a shift in the optical bandgap and PL bands. The photoluminescent properties of these structures may be suitable for the development of new photodetectors and light-emitting devices.

## 2. Materials and Methods

**Figure 1:**
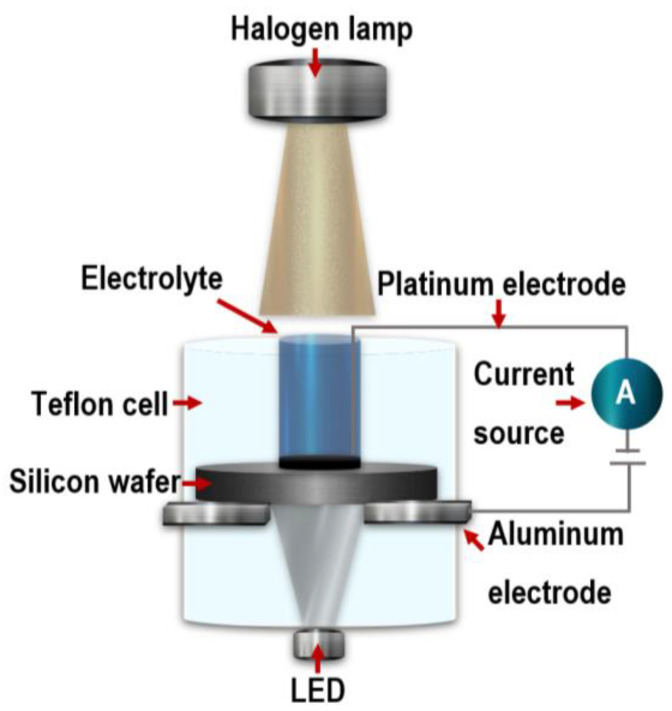
Schematic diagram of the electrochemical cell.

The PSi layers were prepared on n-type silicon substrates with (1 0 0) orientation and low resistivity (5 × 10^−3^ Ω∙cm) by electrochemical anodization. Before electrochemical etching, the substrates were subjected to an MOS cleaning process [17]. The substrates were placed in a Teflon cell with a platinum electrode (cathode) and an aluminum electrode (anode). The etching solution contains C_2_H_6_O:HF:C_3_H_8_O_3_ in a volume ratio of 6:3:1. The etching process was carried out for 120 s, 240 s, 360 s, and 480 s, respectively, with an anodization current density of 75.75 mA/cm^2^, applied over an area of 1.32 cm^2^. A halogen lamp was used as the illumination source to generate the necessary holes and form the pores on the work surface of the silicon wafer, and an LED was used on the backside of the substrate. The electrochemical system is shown in Figure 1.

The CNTs and GO were provided by the Plasma Processing Laboratory, Department of Chemical Engineering, McGill University, Canada. The properties of the CNTs used in this work are described herein [18]. The CNTs were deposited using a spin coating method. According to the reported in the literature [19], due to the low molarity, all the colloidal solutions to be deposited (CNTs and GO) were dissolved in methanol to avoid aggregation of the particles in the solution. The concentrations of the CNTs and GO in the colloidal solutions were 3.6 g/L and 2.6 g/L, respectively. The parameters of the spin coating method used for thin film deposition were as follows: three drops of colloidal solution were added to the PSi, substrates were spun at 700 rpm for 10 s, then at 3000 rpm, for 15 s. This process was repeated three times.

After CNTs and GO deposition, we confined them within the SiP layers, SRO films were deposited on the Si-n/PSi/CNTs and Si-n/PSi/GO structures. The SRO films were deposited using an HFCVD reactor. These depositions were performed at a constant molecular hydrogen (H_2_) flow at 100 sccm level, and the source-to-substrate distance (ssd) was kept at 8 mm. Some parameters were set during the deposition, such as the filament-to-source distance (fsd), which was kept constant at 6 mm, deposition time was 1 min, and the applied voltage to the filaments was 84 V at atmospheric pressure. Table 1 lists the nomenclature used to identify the twelve samples according to the etching time.

The morphology of the PSi was studied by high-resolution scanning electron microscopy (SEM) equipped with an electron field emission source FESEM 7601 (Jeol, Tokyo, Japan) operating at 5 kV of accelerating voltage. The PL spectra of the Si-n/PSi, Si-n/PSi/CNTs, Si-n/PSi/GO, Si-n/Psi/CNTs/SRO, and Si-n/PSi/GO/SRO structures were obtained by using a FluroMax 3 spectroflouorometer (Horiba. Ltd., Kyoto, Japan) with a 150 W xenon excitation lamp, and a high sensitivity emission detector using the following parameters: 1 nm resolution, 370 to 1000 nm range, and the excitation wavelength used was 330 nm. 

## 3. Results and Discussion

**Figure 2:**
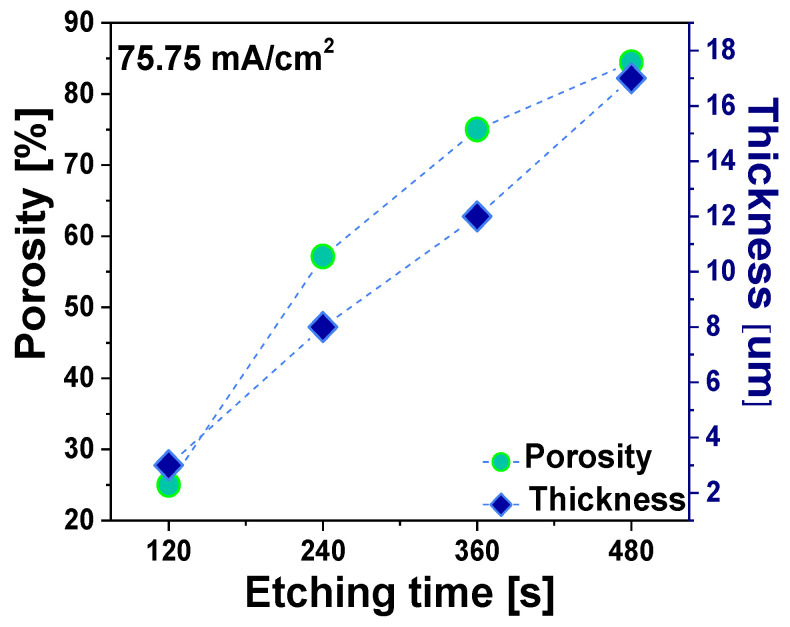
Porosity and thickness as a function of the etching time.

The porosity and thickness of the PSi layers were obtained through gravimetric measurements according to Equations (1) and (2) [20]:

(1) $$\%P=\frac{m_{1}-m_{2}}{m_{1}-m_{3}}\times 100,\;\;\;\;\;$$

(2) $$Thickness=\frac{m_{1}-m_{3}}{\rho \;A},\;\;\;\;\;$$

where *m*_1_ is the weight of the sample before the electrochemical etching, $m_{2}$ is the weight after the formation of PSi, and $m_{3}$ is the weight after the PSi layer removal. The PSi layer thickness was calculated using Equation (2), where *A* is the etching area (1.23 cm^2^) and *ρ* is the silicon density (2328 mg/cm^3^). Figure 2 shows a plot of the PSi porosity and thickness as a function of etching time.

From Figure 2, we observe that the porosity of the PSi layers (increases with etching time) shows a non-linear dependence as function of the etching time. The thickness of the PSi layer increases with longer etching times, and it shows a linear function of etch time. In addition, the etching rate is calculated by the relation r = d/t, where *d* is the thickness of the PSi layer and *t* is the processing time. The etching rates were 25.33 nm/s, 33.33 nm/s, and 35.41 nm/s, respectively. It has been reported that the refractive index (n) of the PSi decreases with increasing porosity; in our case, the values of n are 2.8, 1.7, 1.5, and 1.4, respectively [21,22]. In a previous work, the value of n of the SRO was reported, which was 1.02, and it was obtained by null ellipsometry [10]. The reported values of n for CNTs and GO lie in a range of 1–1.8 and 2.4 and 3, respectively [23,24].

**Figure 3:**
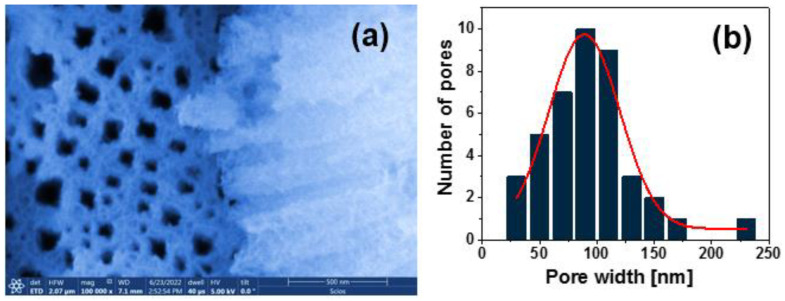
(**a**) SEM micrograph of the A4 PSi layer; (**b**) average size distribution of the pores obtained by particle counting.

To determine the size and distribution of CNTs and GO in methanol solutions, a Zetasizer NanoSeries equipment (Malvern Panalytical. Ltd, Malvern, United Kingdom) was used in dynamic light scattering mode. It was determined that a volume of 5 mL of CNTs/methanol solution in a glass tube contains CNTs with a diameter of 384 nm, whereas the graphene nanoparticles in the GO/Methanol solution their diameters were 157 nm. The results of the morphological characterizations are shown in Figure 3a,b. Figure 3a shows a SEM micrograph showing how the pores join together to form larger pores. This enlargement is due to the pore structure beginning to collapse on each other as this sample was subjected to a more aggressive attack on the silicon surface due to the etching time used. Figure 3b shows that the average size of the pores is about 89 nm, calculated by Image J software.

The presence of different pore geometries in the SiP layer is observed. Since these pores are not circular but have irregular shapes, the present structures can be defined as macroporous with diameters greater than 50 nm, as seen in Figure 3b. Several investigations confirmed that the porosity of the PSi layer increases with etching time [18], as shown in Figure 2.

**Figure 4:**
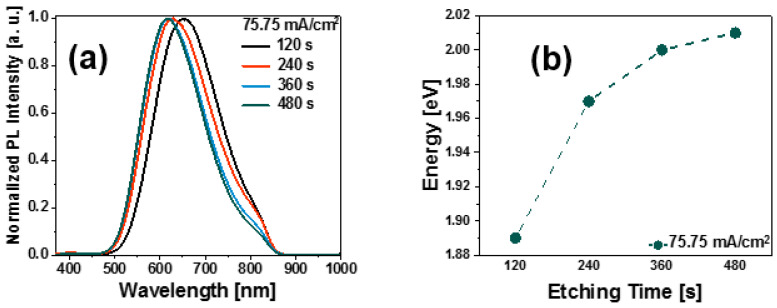
(**a**) Normalized PL spectra of the PSi layers varying the etching time for a constant anodizing current, (**b**) The PL shift peaks of the PSi layers.

The photoluminescent properties of the PSi layers were analyzed before and after the deposition of CNTs and GO. Figure 4a shows the normalized PL spectra of the PSi layers obtained at different etching times. To compare the emission response, all PL spectra were normalized.

From the graphs shown in Figure 4a, we can see a Gaussian-shaped PL bands, located within the approximated wavelength range of 500 nm to 850 nm; in addition, the spectra exhibit a slightly pronounced shoulder, which is weakly noticeable in the 750–850 nm range. It is observed that the PL spectra show a slight shift to shorter wavelengths as the etching time increases. This last property is confirmed by the graphical representation of the PL peaks with their corresponding energies, as shown in Figure 4b. Figure 4b shows the shift of the PL peaks for different etching times of the PSi layers as a function of the band gap energies $E_{g}$. We can observe a shift to higher energies of the PL emission peaks as a consequence of variations in the size of the pores. From the PL spectra, the band gap $E_{g}$ values of the SiP layers were obtained using Equation (3) [25,26].

(3) $$E_{g}=\frac{hc}{\lambda }\;,$$

where *h* is the Plank’s constant, *c* is the speed of light in vacuum, and λ the wavelength in nm. Table 2 lists the calculated values of $E_{g}$ of the PSi layers.

**Figure 5:**
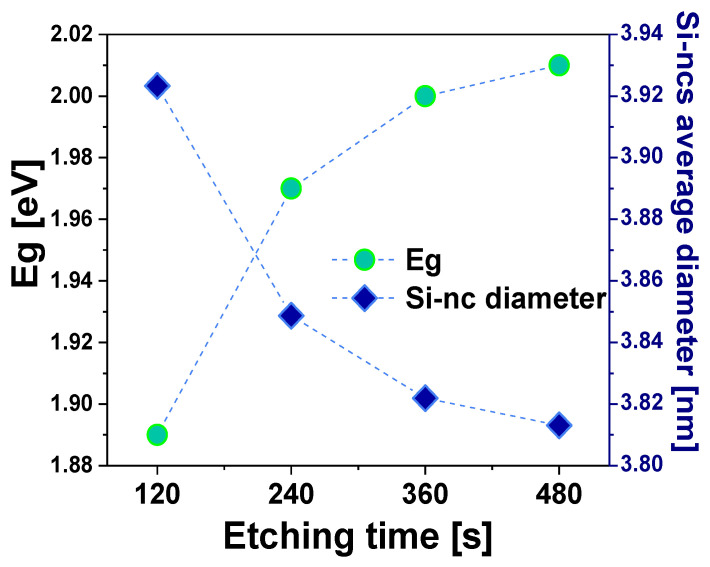
$E_{g}$ and Si-ncs average size of the SiP layers vs. etching time.

The $E_{g}$ values of the PSi determined by Equation (3) lie between 1.89 eV and 2.01 eV, as can be seen in the listed data of Table 2, which are higher than the Si reference value (1.17 eV). In addition, it is observed that $E_{g}$ increases with the etching time, as Figure 5 shows. The increment of the band gap can be attributed to both the quantum confinement effects and defects [27].

Since the nanopore can be considered a quantum dot [25] due to its low dimensions, it is known that in such systems, the discrete electronic energy levels are far apart; thus, the electron may have direct transitions that produce light emission with high energy higher than that of the band gap of the material where they are embedded. Additionally, these energies can be associated with the origin of the PL emission in the PSi. When the pore size is smaller, the emission peak shifts to shorter wavelengths. This effect corresponds to the porosity obtained by gravimetry, as seen in Figure 2, whereby increasing the anodizing time, the PSi layer becomes more porous, indicating an increase in the porosity density and a reduced pore average size. It is possible to estimate the size of the Si-ncs using the effective mass theory. The average nanocrystal size *d* (diameter) may be calculated with Equation (4) [28,29].

(4) $$E\left(eV\right)=E_{g}+\frac{h^{2}}{8d^{2}}\left[\frac{1}{m_{e}^{*}}+\frac{1}{m_{h}^{*}}\right],$$

where *E*(*eV*) is the PL peak position of the PSi, $E_{g}$ is the energy band gap of the Si bulk (1.12 eV), *h* is Planck’s constant (4.13 × 10^−15^ eV·s), m_e_^*^ = 0.19 m_0_, m_h_^*^ = 0.16 m_0_, and m_0_ = 9.1 × 10^−31^ kg. The obtained sizes of the Si-ncs are shown in Figure 5. 

**Figure 6:**
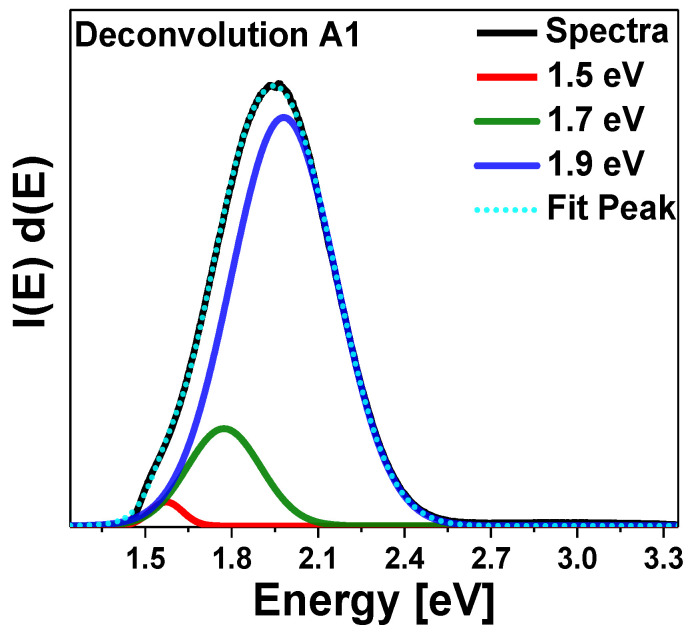
PL spectrum deconvolved for the A3 layer.

The reduction in the nanometric sizes leads to a significant change in the physical properties of Si-nc. This reduction leads to the charge carrier quantum confinement. The transition from indirect to direct band gap occurs when the nanostructures form is dominated by porosities, surface passivation, pore size, and size distribution [30]. For the Psi, the two most accepted phenomena to explain the emission of light in the red and infrared range are as follows; the existence of the quantum confinement effect in the Si-ncs and the presence of defects. In the first phenomenon, the light emission of Si-ncs could be due to the band-to-band radiative recombination process of electron-hole pairs (excitons) confined within the Si-ncs [31]. In the second one, the PL of the Psi layers is due to the presence of defects in the SiO_2_ matrix related to oxygen vacancies or defects at the SiO_2_/Si-ncs interface [32,33,34]. In this context, the emission mechanisms are due to the defects formed during the anodization process. As can be seen in Figure 4a, the PL spectra of Psi are quite broad, so different phenomena occur together in the silicon nanostructure, so several emission mechanisms are probably involved. The interfaces in the Psi (Si/Air, Si/SiO_x_) can perform a crucial role in PL emission. Therefore, to adequately interpret the PL spectra and identify all possible contributions to the Psi emission phenomena, the deconvolution of each spectrum was performed along with an analysis of the identified emission peaks. To perform the deconvolution and avoid common errors reported in the PL analysis, the spectra were deconvolved using the PL intensity-energy differential vs. energy [35]. For this purpose, in Figure 6, as an example, the deconvolution of the PL spectrum is presented corresponding to sample A3.

This figure shows an example of the deconvolution of all other PL spectra of the Psi layers, where, due to the scale of the spectrum, it allows more detailed observation of the deconvolved peaks. The energy position of the peaks obtained from all the deconvolution carried out of the PL spectra are presented in Table 3.

From Table 3, we observe the presence of an emission band, the slow band (S), which is positioned in the yellow-red range. All the PSi layers show the presence of the S-band in which three peaks are present; the first band is in a range from 1.98 eV to 2.09 eV. This band is called the hot band (HB). The HB is due to quantum confinement in Si-ncs and direct band transitions [36,37]. The second peak is identified in the range from 1.77 eV to 1.87 eV, and that emission is associated with luminescent centers located at the Si/SiO_2_ interface [38]. Finally, the third peak appears at 1.57 eV; this one originated by quantum size effects owing to the nanometric dimensions of the filaments forming the PSi layer [39], which can be interpreted as an enlargement of the pores.

**Figure 7:**
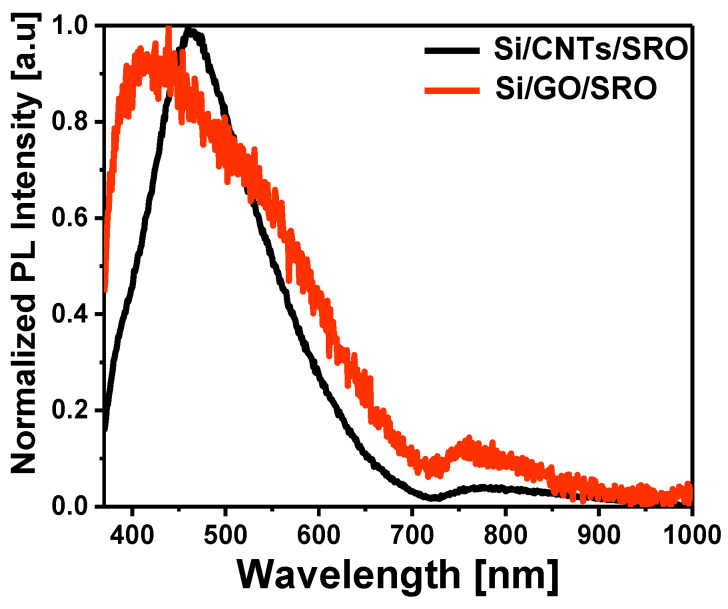
The normalized PL spectra of the Si-n/CNTs/SRO and the Si-n/GO/SRO structures.

Now, we consider the PL spectra of the structures. Figure 7 shows the normalized PL spectra of the Si-n/CNTs/SRO and the Si-n/GO/SRO structures. These samples are without PSi considering that it was necessary to see the contribution of CNTs, GO, and SRO in the complete structure. The PL spectrum of the Si-n/CNTs/SRO structure exhibits a strong and well-defined peak in the blue band (2.7 eV), which is attributed to the CNTs, and there is also a peak of lower intensity in the red band around 1.6 eV. Two bands appeared in the Si-n/GO/SRO structure, one of which is well-defined at 2.9 eV, and it is attributed to the GO, just as in the structure with the CNTs. The other peak of low intensity appears around 1.6 eV, and it is a characteristic peak of the SRO films [7].

The broad PL spectrum suggests that multiple emission mechanisms may be involved. To find out the contributions to the PL spectra, these ones were deconvolved. The different peaks defined by the deconvolution are associated with different types of defects with their respective energy positions, as seen in Table 4.

Table 4 shows the emission mechanisms associated with the CNTs, the GO, and the SRO. For the CNTs, two emissions were identified, one of them is in the blue region at 2.75 eV and the other emission is in the green region. Similarly, GO has two emissions peaks: the first peak is located in the violet region (3 eV), and the other one is in the green region (2.5 eV). Finally, a peak associated with non-bridging oxygen hole centers (NBOHC) was identified for silicon-rich oxide (1.6 eV). In the structures with the CNTs and GO, both a PL shift and a stronger emission are observed. In the case of the CNTs structure, the curvature effects and interactions between the walls of the nanotube, and the edge effects and chiral indices (n,m), significantly modify their electronic structure, favoring the electronic transitions and, consequently, the radiation emission [44,45].

**Figure 8:**
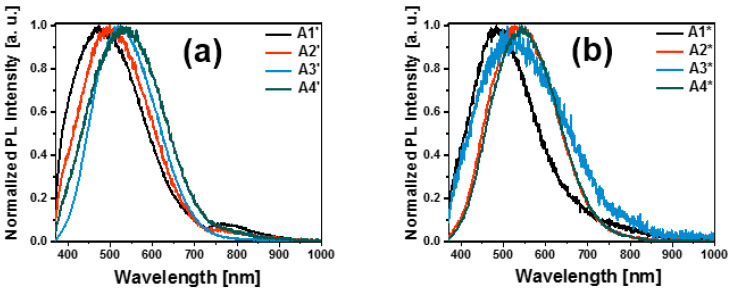
(**a**) The normalized PL spectra of the Si-n/PSi/CNTs/SRO structure, (**b**) normalized PL spectra of the Si-n/PSi/GO/SRO structure.

Figure 8a,b show the normalized PL spectra of the Si-n/PSi/CNTs/SRO and Si-n/PSi/GO/SRO structures, respectively. The PL spectrum of the Si-n/PSi/CNTs/SRO structure shows a strong and well-defined peak in blue (2.8 eV), which is attributed to the CNTs; in addition, a peak of lower intensity is located in the red band around 1.7 eV. For the Si-n/PSi/GO/SRO structure, a more defined peak around 2.5 eV is observed, which is attributed to the GO. Similar to the structure with the CNTs, a peak of low intensity appeared around 1.7 eV, and this is a characteristic peak of the SRO films. In general, the PL spectra of the structures have contributions of the PSi, CNTs, GO, and SRO; therefore, their spectra are very broad.

The spectra shown in Figure 8a,b were deconvolved to determine the emission mechanisms present in the structures. They are detailed in Table 5.

**Figure 9:**
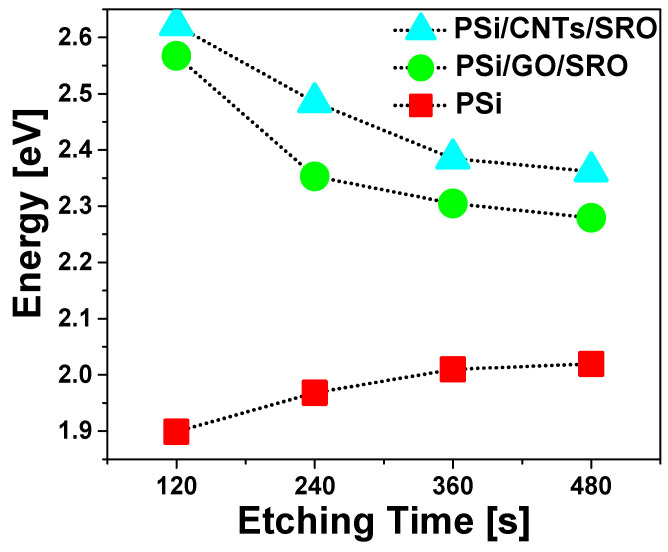
PL shift peaks of PSi layers, Si-n/PSi/CNTs/SRO and Si-n/PSi/GO/SRO structures.

To compare the PL emissions of the structures, Figure 9 shows the PL emission shifts of the structures. For the PSi, it is observed that as etching time increases, the PL emission moves to higher energies, due to quantum confinement effects present in the PSi layer caused by the increase in porosity. However, for the structures with the CNTs and GO, the behavior is opposite. As the etching time increases, the PL emission moves toward lower energies and, the emission present in the structures with the CNTs is more energetic than that corresponding to the structures with GO. 

Blue emission (2.8 eV) exhibited in the Si-n/PSi/CNTs/SRO structures has already been observed in diamond films and it is attributed to luminescent centers present in the CNTs [40]. The green emission (2.5 eV) is due to nitrogen contamination; N-bonded carbons have been reported to act as luminescent centers in the CNTs, such as N-defects in CVD-derived nanodiamonds and in natural diamonds [42]. It is known that CVD-derived CNTs and diamond contain nitrogen and nitrogen-related defects, thus, the spectra suggest that the PL of the samples may have a similar origin based on nitrogen-bonded carbon [42]. On the other hand, the C=O functional groups present on the surface of CNTs has also been reported for the green emission origin [40]. It has been reported that the influence of Si-O, SiH_x_, and Si-OH bonds determines the shape of the PL spectra, and to a lesser extent, the influence of hydrogen-carbon surface created between the carbon atoms in the CNTs and H-Si in the PSi layer [18].

It was observed that the GO exhibits three emission bands located at 2.4 eV, 2.8 eV, and 3 eV, respectively. These bands are similar those reported in the literature [41]. The emission mechanism at 3.1 eV is due to the formation of excimer molecules, which can occur between polyaromatic molecules and conjugated polymers. Similarly, excimers can form the GO sheets through π−π overlap orbitals [41]. The origin of the emission peak located at 2.8 eV is due to the presence of graphene quantum dots (GQD) [46]. It has been reported that nitrogen doping of the GQDs containing oxygen-rich functional groups causes a shift of the PL to the blue region [46]. Similarly, the PL of the GQDs is strongly affected by the π-π* transition, which originated from the nitrogen-containing aromatic rings and graphene structure [47]. The GO contains highly oxidized polycyclic aromatic hydrocarbon fragments, called oxidative debris (OD), which are present in the GO and are attached to the GO in a graphene-like plane [47]. The OD has oxygen-containing groups, such as the GO, so these groups should not be a direct source of the 2.5 eV band. Therefore, it is plausible that the 2.5 eV band is due to the interaction between the graphene plane and the OD. Oxygen-containing groups in the GO, such as carboxyl or epoxy groups, always induce non-radiative electron-hole pair recombination, making the emission intensity of the GO very weak. [48]. The atomic composition of the GO is believed to be a graphene basal plane with a non-uniform distribution of oxygen-containing groups; resulting in sp^2^ carbon clusters that have a few nanometers and are isolated within a defective carbon lattice or the sp^3^ matrix [49]. In carbon materials containing a mixture of sp^2^ and sp^3^ bonds, the optoelectronic properties are mainly determined by the π and π* states of the sp^2^ sites that lie within the σ-σ* gap [44]. Since the π-bond is weaker and has a lower formation energy, as a result, it is predicted that many localized states that are caused by disorder are present in the two-dimensional network of the as-synthesized GO, which has a large percentage of distorted carbon atoms attached to oxygen-containing functional groups. Since the interactions of the π-states depend strongly on their projected dihedral angle, the localized states caused by structural disorder may be in the band’s tail or even deeper within the bandgap [48].

The deposited SRO films show weak emission. However, the observed PL emission has a peak value of about 1.7 eV, proving that the PL emission is not due to interband recombination. Therefore, the most likely mechanism is emission by localized states due to NBOHC [42].

## 4. Conclusions

The Si-n/Psi, Si-n/CNTs/SRO, Si-n/GO/SRO, Si-n/Psi/CNTs/SRO, and Si-n/PSi/GO/SRO structures were performed, and their PL spectra were measured. They were studied according to their PL emission shift and wavelength modulation. The energy of the PL peaks varied according to the type of structure performed. The PL emission of the PSi layers is attributed to quantum confinement and some defects caused by pores. The PL emission of the CNTs was localized in the blue-green region; the blue emission (2.8 eV) is due to luminescent centers associated with diamond films; additionally, the green emission (2.5 eV) is caused by nitrogen bonded carbons. In addition, the green emission is also attributed to C=O functional groups present in the CNTs surface. The GO emission was observed in two bands, 3 eV and 2.4 eV, caused by oxidative debris (OD) and excimers, respectively. Whereas for the SRO film, a contribution at 1.7 eV emission is due to the presence of nonbridging oxygen hole centers defect. In the Si-n/PSi/CNTs/SRO and Si-n/PSi/GO/SRO structures, the PL spectra have contributions from the PSi, CNTs, GO, and SRO, therefore, the PL is very broad and different types of defects linked to the position of the PL peaks were obtained. Structures with the PSi, CNTs, and SRO have great potential in optoelectronics devices applications due to the modulation of the PL emission towards high energies for the structures with the CNTs and GO. In summary, these structures exhibit relevant PL properties, whereby they are promising candidates to be used in the field of optoelectronic devices, such as photodetectors, electroluminescent devices, and solar cells.

## Figures and Tables

**Table 1:** List of etching conditions of the SiP layers, Si-n/PSi/CNTs (´) and Si-n/PSi/GO (*) structures.

75.75 mA/cm^2^ Anodizing Current Density			
Etching Time	Sample	With CNTs	With GO
120 s	A1	A1′	A1*
240 s	A2	A2′	A2*
360 s	A3	A3′	A3*
480 s	A4	A4′	A4*

**Table 2:** Energy values [eV] of the band gap of PSi layers.

75.75 mA/cm^2^ Anodizing Current Density	
Etching Time [s]	Band Gap Energy [eV]
120	1.89
240	1.97
360	2
480	2.01

**Table 3:** PL deconvoluted bands and the position [eV] of the identified peaks.

Sample	S Band		
A1	1.98	1.77	1.57
A2	2.04	1.78	1.57
A3	2.07	1.81	1.57
A4	2.09	1.87	-

**Table 4:** Types of defects linked to the position [eV] of the PL peaks of the structures.

Si-n/CNTs/SRO PL Emission Mechanisms		Si-n/GO/SRO PL Emission Mechanisms	
Luminescent centers associated with diamond films	2.7 [40]	It is attributed to the excimer present in the GO	3 [41]
Defects due to Nitrogen	2.4 [42]	Interactions between graphene planes and OD (Oxidative debris)	2.5 [41]
Non-bridging oxygen hole centers (NBOHC)	1.6 [43]	Non-bridging oxygen hole centers(NBOHC)	1.6 [43]

**Table 5:** Types of defects linked to the position [eV] of the PL peaks of the structures.

**Si-n/PSi/CNTs/SRO PL Emission Mechanisms**				
	**A1′**	**A2′**	**A3′**	**A4′**
Luminescent centers associated with diamond films [40]	2.8	2.8	2.7	2.8
Defects due to Nitrogen [42]	2.4	2.5	2.4	2.5
PSi band gap	2.1	2.2	2.1	2.1
Non-bridging oxygen hole centers [42]	1.7	1.7	1.7	1.7
**Si-n/PSi/GO/SRO PL emission mechanisms**				
	A1*	A2*	A3*	A4*
It is attributed to the excimer present in the GO [41]	3.1	-	-	-
Graphene oxide quantum dots [46]	2.8	2.8	2.8	2.7
Interactions between graphene planes and OD [44]	2.4	2.4	2.4	2.4
Porous silicon band gap	2.1	2.2	2	2.1
Non-bridging oxygen hole centers [42]	1.7	1.8	1.6	1.7

## Data Availability

Not applicable.
